# Current-state opacity verification in discrete event systems using an observer net

**DOI:** 10.1038/s41598-022-25697-y

**Published:** 2022-12-14

**Authors:** Abdeldjalil Labed, Ikram Saadaoui, Naiqi Wu, Jiaxin Yu, Zhiwu Li

**Affiliations:** 1 Institute of Systems Engineering, Macau University of Science and Technology, Taipa, 999078 Macau SAR China; 2 Mediterranean Institute of Technology, South Mediterranean University, 99628 Tunis, Tunisia; 3 Hitachi Building Technology (Guangzhou) Co., Ltd., Guangzhou, 510670 China

**Keywords:** Computer science, Computational science

## Abstract

Due to the proliferation of contemporary computer-integrated systems and communication networks, there is more concern than ever regarding privacy, given the potential for sensitive data exploitation. A recent cyber-security research trend is to focus on security principles and develop the foundations for designing safety-critical systems. In this work, we investigated the problem of verifying current-state opacity in discrete event systems using labeled Petri nets. A system is current-state opaque provided that the current-state estimate cannot be revealed as a subset of secret states. We introduced a new sub-model of the system, named an observer net. The observer net have the same structure as the plant, but it is distinguished by the use of colored markers as well as simultaneous and recursive transition enabling and firing, which offer an efficient state estimation. We considered two settings of the proposed approach: an on-line setting, in which a current-state opacity algorithm is proposed. The algorithm waits for the occurrence of an observable event and determines if the current observation of a plant reveals the secret behaviour, as well as, an off-line setting, where the verification problem is solved based on a state estimator called a colored estimator. In this context, necessary and sufficient conditions for verifying opacity are developed with illustrative examples to demonstrate the presented approach.

## Introduction

Cybersecurity is needed as an extension of reliability to protect systems from errors or damage caused by cyberattacks. Nowadays, ensuring the privacy of information flows^1,2^ has become an increasingly significant concern^3–7^. Formalizing security principles and developing theoretical basics for secure systems design is a current cybersecurity research trend. Specifically, in discrete event systems (DESs), the problem of whether privacy is disclosed in information flows can be addressed in terms of a confidentiality property called opacity. Opacity characterizes whether the secret behaviour of a considered system is revealed to an external observer or intruder. The term opacity is first used to describe cryptographic protocols in^8^ and then expanded to the DES domain through the work of Bryans et al.^9^, where it formally expresses the absence of information flow, i.e., the intruder’s inability to make any useful inference about the secret information in systems modeled as Petri nets (PNs). In DESs, the secret can be represented by the states or language of a system. Accordingly, opacity properties are generally classified into two types: state-based opacity (SBO)^9–11^ and language-based opacity (LBO)^12,13^.

The computer security community has recently looked into several aspects of opacity. A brief overview of some of the recent studies is provided in what follows. In^14^, Yang et al. propose new types of opacity in networked DESs that are modeled as finite state automata (FSA) by taking into account the communication delays and losses in the observation channel. Opacity was also investigated in stochastic DESs modeled as probabilistic automata^15^ and for fuzzy DESs modeled as fuzzy automata^16,17^, which extends the relevant findings of the opacity theory for classical DESs. It should be noted that when a system is evaluated to be non-opaque, opacity enforcement becomes crucial. In this context, opacity enforcement has been considered either by restricting the system behavior using supervisory control^18,19^ or by changing the information flow using insertion function^20,21^.

This paper aims to verify the property of current-state-based opacity (CSO) for DESs that can be described by bounded PNs. It is assumed that an intruder completely knows the system structure, but can partially observe the occurrence of some events only. Therefore, he/she tries to estimate the system states based on its observations to infer the secret behaviour. The system considered in this work is represented by labeled PNs (LPNs) with a static observation function^22,23^. A subset of the reachable markings represents the secret. A system is CSO if an intruder cannot unambiguously discover the secret states from its observations.

The problem of CSO verification is shown to be decidable for bounded labeled Petri nets^24,25^. However, Tong et al.^26^ recently demonstrate that, in general, the opacity verification problem is undecidable if the PN system is unbounded. For this reason, our work concentrates on bounded LPN by proposing an efficient approach that provides definite answers to the CSO problem.

Many of the existing studies on DESs pay particular attention to the opacity problem. Various methods have investigated the issue of state-based opacity verification in DESs^27,28^. In^10^, the authors report a necessary and sufficient criterion using a non-deterministic finite automaton (NFA) by building an observer, i.e., transforming an NFA into a deterministic finite automaton (DFA) with a complexity of ${\mathcal {O}}(2^n)$^29^, where *n* is the number of states in the NFA. However, the verification of CSO is proved to be PSPACE-complete with respect to *n*^30–32^. By using a compact representation of a reachability graph (RG) called a basis RG (BRG), the work in^27^ presents a necessary and sufficient condition for CSO. Note that the concept of BRGs have been proposed in^33–36^. The benefit of this method consists in avoiding the exhaustive enumeration of all reachable markings. However, the computational effort is still considerably heavy, and a large amount of memory is required.

Another interesting work is recently presented in^28^, where the authors discuss CSO modeling and verification in DESs modeled by partially observed PNs (POPNs)^37^. They propose a discernible reachability graph (DRG) to compute the state estimation of a POPN system and check if the opacity condition holds. Its limitation lies in the fact that the DRG alone does not provide a necessary and sufficient CSO verification condition. Consequently, the authors resort to integer linear programming (ILP) to solve this problem. In the same context, online verification algorithms for current^38^ and initial^39^ state opacity have been proposed by Cong et al. in LPNs by assuming the acyclicity of the observable and unobservable subnets. These algorithms detect the occurrence of events and decide whether the transition (event) sequence observed so far is opaque or not. This decision is based on solving a group of ILPs. The works in^38,39^ are restricted to secret markings defined by generalized mutual exclusion constraints (GMECs)^40^.

On the other hand, LBO has been formalized in the existing studies in various ways. It is first proposed in the framework of NFA^41,42^. The secret for the LBO problem is described by a sub-language of the DES. A system is said to be of LBO with respect to a secret language if an intruder cannot reveal that any generated event sequence is entirely within the secret. In^43^, the authors characterize and introduce two types of opacity on the basis of languages, namely strong opacity and weak opacity. In^12^, the authors propose approaches to ensure language-based opacity for bounded LPNs based on finite-time automata, called a verifier, by assuming that an intruder captures observable transitions only. For LBO verification using ILP, the work in^44^ formulates a necessary and sufficient condition. Jacob et al. provide a thorough overview of opacity for DESs^31^. A historical perspective on the development of the opacity theory (and diagnosability theory) in DESs can be found in^45^.

This work investigates CSO using a new model called observer net. The main contributions of this work can be summarized below:

1. A new sub-model of the system called an observer net is developed based on the plant structure. It is characterised by the new concepts of simultaneous and recursive transition enabling and firing allowing a rapid computation of the reachable markings.
2. We proposed an on-line algorithm for CSO verification in an LPN system. It provides the state estimation and the opacity decision of the word observed so far by waiting the occurrence of an event and then determines if the last observed event reveals the secret behaviour or not.
3. The proposed observer net model provides efficient usage of space, while improving runtime performance. We managed to lower the space complexity by avoiding the exhaustive computation of all reachable markings, and also lower the time complexity by merging the computation phases using the new concepts of simultaneous and recursive transition enabling and firing.
4. When an off-line opacity verification is desired, we constructed a state estimator called a colored estimator, where each of its states corresponds to a set of the consistent markings.

The remainder of this paper is structured as follows. In section “Current-state opacity”, we state the problem of CSO and present its definitions. Section “Observer net” introduces the concept of the observer net and specifies its dynamics. In section “Verification of current-state opacity”, we verify current-state opacity using on-line and off-line algorithms. Section “Computational complexity and comparison” investigates the proposed approach’s effectiveness by giving a comparative study with related works. In section “Conclusions”, concluding remarks and possible future directions are discussed.

## Current-state opacity

We intend to define the notion of opacity in a DES modeled as a PN. In a system modeled with an LPN $G=(N, M_{0}, E, \ell )$, a secret *S* is a subset of the reachability set composed of some particular markings, called secret markings. Current-state opacity claims that, for every secret state reachable from the initial state by firing a transition sequence, a non-secret state reachable by firing another transition sequence must exist, and both sequences have the same observation from the intruder perspective. Moreover, it is assumed that an intruder knows the system’s structure, but he/she can get a partial observation of the event occurrences only. Necessary preliminaries are provided in the appendix of this study^46^.

### Definition 1

^27^ Given an LPN $G=(N, M_{0}, E, \ell )$ and a secret $S \subseteq R(N,M_{0})$, we say that observation $w \in {\mathcal {L}}(N,M_{0})$ is $\text {current-state opaque}$ wrt *S* if ${\mathcal {C}}(w) \nsubseteq S$ holds.

### Definition 2

^27^ We say that $G = (N,M_{0}, E, \ell )$ is $\text {current-state opaque}$ wrt $S \subseteq R(N,M_{0})$ if for any $w \in {\mathcal {L}}(N,M_{0})$, we have ${\mathcal {C}}(w) \nsubseteq S$.

Namely, for any possible $w \in {\mathcal {L}}(N, M_0)$, an intruder is unable to determine if the current state lies within *S*. Now, we define the non-current-state opaque observation and system as follows.

### Definition 3

^39^ Given $G = (N,M_{0}, E, \ell )$ as an LPN system and $S \subseteq R(N,M_{0})$ as a secret, if ${\mathcal {C}}(w) \subseteq S$ holds, then $w \in {\mathcal {L}}(N, M_{0})$ is non-current-state opaque wrt *S*.

For a non-CSO observation *w*, an intruder can deduce that any marking consistent with *w* is within *S*, i.e., for any $M \in {\mathcal {C}}(w)$, $M \in S$. Accordingly, a non-current-state opaque system is defined as follows.

### Definition 4

^39^ We say that an LPN $G = (N, M_{0}, E, \ell )$ is $\text { non- current-state}$ $\text {opaque}$ wrt a secret $S \subseteq R(N,M_{0})$ if there is at least an observation $w \in {\mathcal {L}}(N, M_{0})$ with ${\mathcal {C}}(w) \subseteq S$.

Based on Definition 4, to ensure the CSO of a bounded LPN system, we need to check whether there is at least a $w \in {\mathcal {L}}(N,M_0)$ such that ${\mathcal {C}}(w) \subseteq S$. To answer this question, one must perform an exhaustive enumeration of all reachable markings, i.e., computing ${\mathcal {C}}(w)$ for all $w \in {\mathcal {L}}(N,M_0)$, and then build a reachability graph observer, i.e., a DFA equivalent to the RG, using the standard determinization procedure^47^, whose computational complexity is ${\mathcal {O}}(2^{|X|})$ with *X* being the set of states in the RG^31,47,48^. The reachability graph observer provides the state estimation after the occurrence of an observation sequence as shown in the following example.

**Figure 1:**
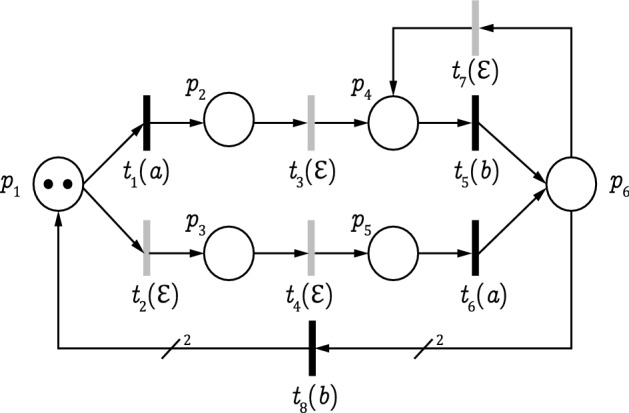
An LPN with $M_{0}=2p_1$.

**Figure 2:**
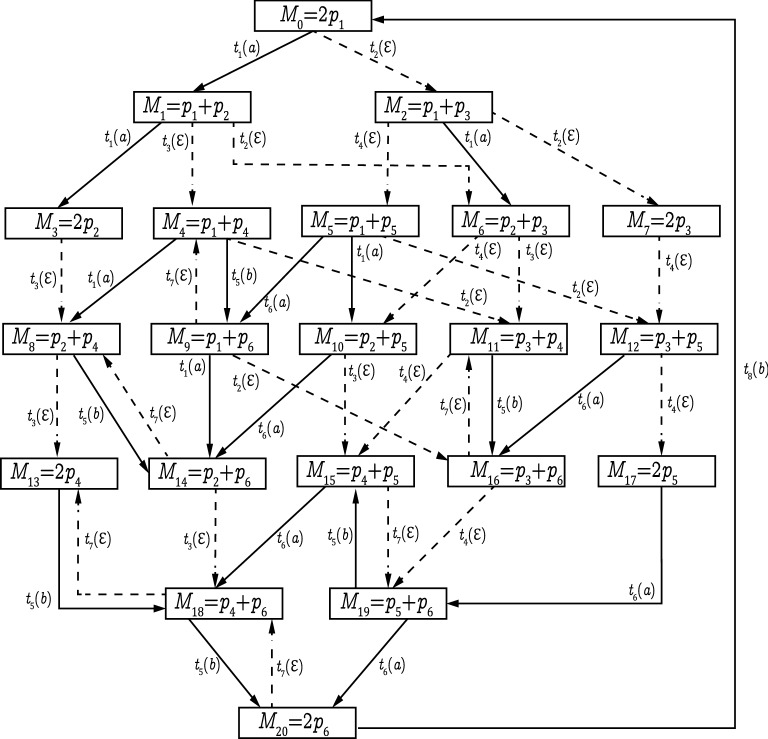
RG of the LPN in Fig. 1.

**Figure 3:**
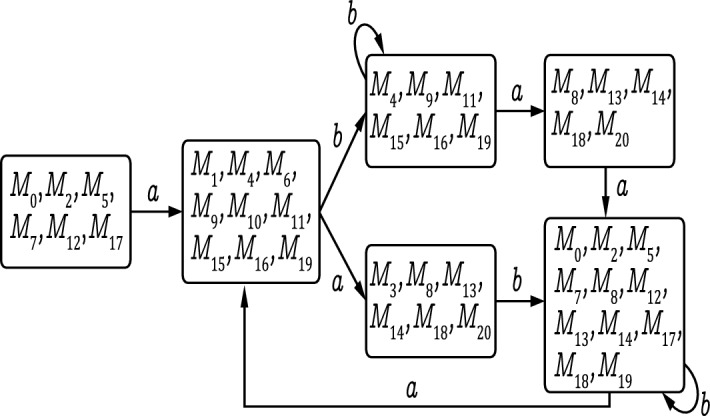
Observer of the RG in Fig. 2.

### Example 1

We consider the plant G in Fig. 1 with initial marking $M_0 = 2p_1$ and $E = \{a, b\}$. The sets of unobservable and observable transitions are $T_{uo}=\{t_{2},t_{3},t_{4},t_{7}\}$ and $T_{o}=\{t_{1},t_{5},t_{6},t_{8}\}$, respectively. The RG and its corresponding observer are given in Figs. 2 and 3, respectively. Let $S = \{M_{8},M_{13},M_{14},M_{18},M_{20}\}$ be a secret. For observation $w=aba$, we have ${\mathcal {C}}(w) =\{ M_8,M_{13},M_{14}, M_{18},M_{20}\} \subseteq S$. Then, based on Definition 4, the LPN system *G* is non-CSO with respect to *S*.

## Observer net

**Figure 4:**
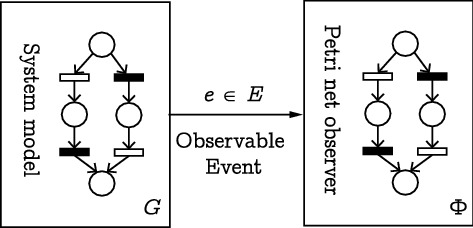
Observer net.

This section defines the concept of an observer net. For a plant *G*, an observer net is a labeled Petri net that has the same structure of *G* (in terms of places, transitions, and arcs) but has a different behaviour. Specifically, an observer net allows the simultaneous presence of several markings, characterised with distinct colors, in order to determine the states the plant can be in upon observation of an event. In Fig. 4, we summarize the interaction between a plant *G* and its associated observer net $\Phi$. Upon the occurrence of an event, the observer net $\Phi$ determines the system state estimation. Specifically, it finds the possible marking at which a plant may lies, i.e., all the states consistent with the sequence of events observed thus far.

The primary challenges in this work lie in defining how the observer net is modeled, graphically represented, and how it operates. Although the observer net is modeled as a labeled Petri net graphically, its state transition function and states differ from regular Petri nets. In what follows, a formal definition of the observer net, and its construction algorithm, as well as its dynamics, are presented.

Note that, in the following, the word “marking” refers to a marking of the plant and the word “state” refers to a marking of the observer net.

### Definition 5

For an LPN $G = (N, M_{0}, E, \ell )$, we define its associated observer net as a six-tuple $\Phi =(N, M_{\Phi ,0}$, *E*, $\ell$, ${\mathcal {C}}_c$, ${\mathcal {C}}_M)$, where

1. ${\mathcal {C}}_c$ is a non-empty and finite set of colors.
2. ${\mathcal {C}}_M:$ $R(N,M_{0}) \rightarrow {\mathcal {C}}_c$ is a function for associating each marking $M \in R(N,M_{0})$ with a color $c \in {\mathcal {C}}_c$.
3. $M_{\Phi ,0}=\{(M,c)| M \in {\mathcal {U}}(M_0) \text { and } {\mathcal {C}}_M(M)=c\in {\mathcal {C}}_c\}$ gives the initial state of the observer net.

The structure of $\Phi$ is same as that of the plant *G*. Its initial state $M_{\Phi ,0}$ consists of colored markings (*M*, *c*), where $M \in {\mathcal {U}}(M_{0})$ and *c* is generated by the function ${\mathcal {C}}_M$. A state in $\Phi$ is a set of colored markings (*M*, *c*), denoted as $M_{\Phi }$, specifying the system state estimation after observing an event. We need to make sure that ${\mathcal {C}}_M$ associates distinct colors to the markings belonging to $M_{\Phi }$ to tell the distinction between them (due to the simultaneous presence of different colored markings in the observer net, it can be thought of as being a special class of colored Petri net).
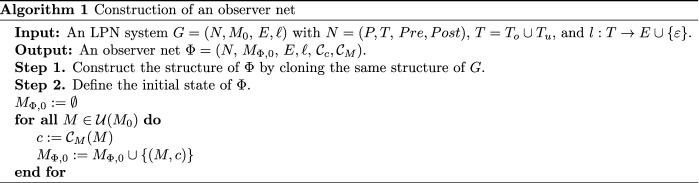


Algorithm 1 takes an LPN $G = (N,M_{0}, E, \ell )$ as input and outputs its associated observer net $\Phi = (N,$ $M_{\Phi ,0},$ $E, \ell ,$ ${\mathcal {C}}_c, {\mathcal {C}}_M)$. In the first step, we build the structure of the observer net $\Phi$ by cloning the plant *G*, i.e., *G* and $\Phi$ have the same structure $N=(P, T, Pre, Post)$, and the same labeling function $\ell$. Then step 2 defines the initial state $M_{\Phi ,0}$ of $\Phi$ by calculating the unobservable reach of the initial marking $M_0$ of *G*, and assigns a distinct color $c \in {\mathcal {C}}_c$ to each marking using the color function ${\mathcal {C}}_M$. This step runs iteratively until all the unobservable reaches of $M_0$ are colored. The computational complexity of Algorithm 1 is mainly dependent on the number of markings in the unobservable reach of $M_0$.

**Figure 5:**
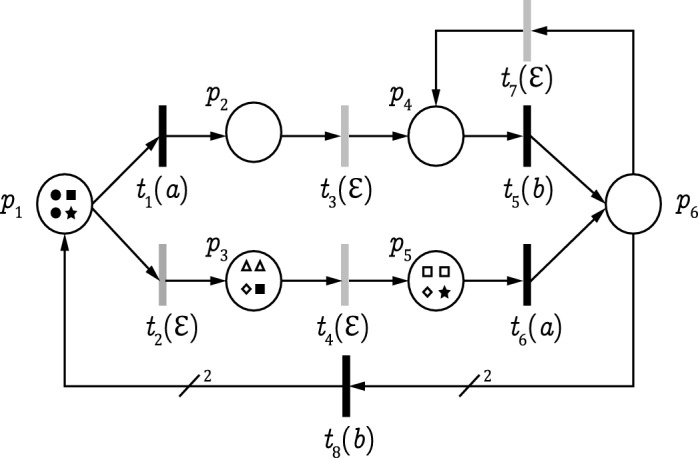
Observer net $\Phi$ with an initial state $M_{\Phi ,0}$.

### Example 2

Let us consider the LPN system *G* in Fig. 1. According to Algorithm 1, the observer net $\Phi$ is shown in Fig. 5 it has the same structure (states and transitions) as plant *G*. The initial state of $\Phi$ is retrieved from the initial marking of *G*. We have $M_0 = 2p_1$ and ${\mathcal {U}}(M_0) = \{ M_0,M_2,M_5,M_7,M_{12},M_{17}\}$; based on Definition 5. the initial state of the observer net is given by:

$$\begin{aligned} \begin{aligned} M_{\Phi ,0} = \{(M_0,\bullet ),(M_2,\blacksquare ),(M_5,\bigstar ), (M_7,\triangle ),(M_{12},\diamond ), (M_{17},\square )\} \end{aligned} \end{aligned}$$

**Figure 6:**
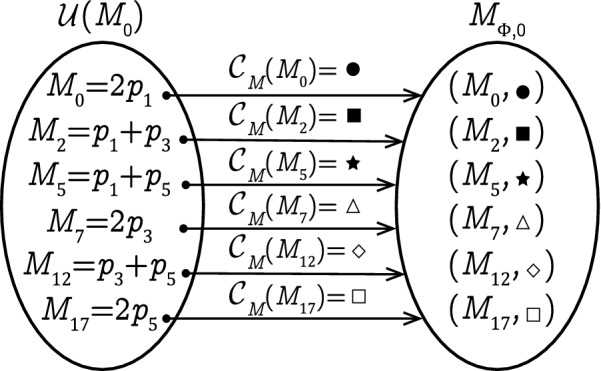
The initial state $M_{\Phi ,0}$ of the observer net.

The initial state of the observer net is composed of six colored markings as shown in Fig. 6.

The dynamic behaviour of a PN is characterized by the transition firing rules together with the distribution of tokens in places. In the following, we introduce the rules that govern the flows of tokens in the observer net.

Given an observer net $\Phi =(N,M_{\Phi ,0},$ *E*, $\ell$, ${\mathcal {C}}_c$, ${\mathcal {C}}_M)$ and a state $M_{\Phi }$, a transition $t \in T_{o}$ is enabled at $M_{\Phi }$ if there exists $(M, c)\in M_\Phi$ such that $M\ge Pre(t,\cdot )$ holds, and we denote it by $(M, c)[t\rangle$. The set of enabled transitions at (*M*, *c*) with label *e* is defined as

1 $$\begin{aligned} \Sigma ((M,c), e)=\{ t \in T_o| (M,c)[t\rangle ~\text {and}~\ell (t)=e\} \end{aligned}$$

and the set of all the enabled transitions at $M_{\Phi }$ with label *e* is defined as

2 $$\begin{aligned} \Sigma (M_{\Phi }, e)=\bigcup _{(M,c) \in M_{\Phi }} \Sigma ((M, c) ,e) \end{aligned}$$

### Example 3

Consider the LPN *G* in Fig. 1, its corresponding observer net $\Phi$ is visualized in Fig. 5 with initial state is $M_{\Phi ,0}$ $= \{(M_0,\bullet ),$ $(M_2,\blacksquare ),$ $(M_5,\bigstar ),$ $(M_7,\triangle ),$ $(M_{12},\diamond ),$ $(M_{17},\square )\}$. Based on Equation (1), we have:

$$\Sigma ((M_0,\bullet ), a) =\{t_1\},$$ $$\Sigma ((M_2,\blacksquare ),a) =\{t_1\},$$ $$\Sigma ((M_5,\bigstar ), a) =\{t_1,t_6\},$$ $$\Sigma ((M_7,\triangle ), a) =\emptyset ,$$ $$\Sigma ((M_{12},\diamond ), a) =\{t_6\},$$ and $$\Sigma ((M_{17},\square ), a) =\{t_6\}$$.

Based on Equation (2), the set of enabled transitions at $M_{\Phi ,0}$ with label *a* is given by:

$$\begin{aligned} \begin{aligned} \Sigma (M_{\Phi ,0}, a) ={}&\bigcup _{(M,c)\in M_{\Phi ,0}} \Sigma ((M,c),a) = \{{t_{1},t_{6}}\} \end{aligned} \end{aligned}$$

Firing a transition $t \in \Sigma ((M,c), e)$ at $(M,c)\in M_\Phi$ yields a new colored marking $(M',c')$, denoted as $(M, c)[t\rangle (M',c')$. We define by

3 $$\begin{aligned} \begin{aligned} \eta ((M,c), e) = \left\{ (M',c')| \exists t\in \Sigma ((M,c), e): \right. \left. (M,c)[t\rangle (M',c') \text { and } {\mathcal {C}}_M(M')=c'\right\} \end{aligned} \end{aligned}$$

the set of reachable colored markings if all enabled transitions in $\Sigma ((M,c), e)$ are fired, and by

4 $$\begin{aligned} \eta (M_{\Phi }, e)=\bigcup _{(M,c)\in M_{\Phi }}\eta ((M,c), e) \end{aligned}$$

the set of all reachable colored markings if the enabled transitions at state $M_{\Phi }$ with label *e* are fired.

Note that, if a transition with label *e* fires at a colored marking (*M*, *c*), all the enabled transitions with label *e* at (*M*, *c*) fire concurrently. Thus, the semantics of an observer net is different from the classical Petri nets.

The following rules define the dynamics of an observer net.

- **Rule 1: Simultaneous enabling** A set of *k* transitions $\{t\in T_{o}\mid \ell (t)=e\}$ are simultaneously enabled at a colored marking (*M*, *c*) if any $t\in {T_{o}}$ with $\ell (t)=e$ is enabled at (*M*, *c*).
- **Rule 2: Simultaneous firing** Simultaneously firing *k* enabled transitions $t_1$, $t_2$, $\ldots$, $t_k \in {T_{o}}$ with label *e* at (*M*, *c*) yields *k* colored markings, as defined by $\eta ((M,c),e)$ in (3).
- **Rule 3: Recursive enabling** A $t\in {T_{o}}$ with $\ell (t)=e$ is said to be recursively enabled at a state $M_\Phi$ if there exist *k* colored markings $\{(M_1,c_1),$ $(M_2,c_2)$, $\ldots , (M_k,c_k)\}$ in $M_\Phi$, for all $i\in \{1,2,\ldots ,k\}$, $(M_i,c_i)$ enables *t*.
- **Rule 4: Recursive firing** The firing of a recursively enabled transition $t \in {T_{o}}$ with label *e* at $M_\Phi$ is defined by firing *t* at $(M_1,c_1)$, $(M_2,c_2)$, $\ldots , (M_k,c_k)$, respectively. Equivalently, *t* fires *k* times, yielding *k* colored markings as defined by $\eta (M_{\Phi },e)$ in (4).
- **Rule 5: Irrelevant markings elimination** If a colored marking $(M,c) \in M_\Phi$ does not enable any transition $t \in {T_{o}}$ with label *e*, i.e., $\Sigma ((M,c),e)=\emptyset$, then, for (*M*, *c*), let $M(p)=0$ for all $p\in P$.

The observer net dynamic rules allow us to benefit from the simultaneous and recursive firing mechanism, which ensures a rapid computation of markings and guarantees a significant decrease in the time complexity of the proposed method, as shown in the following sections.

**Figure 7:**
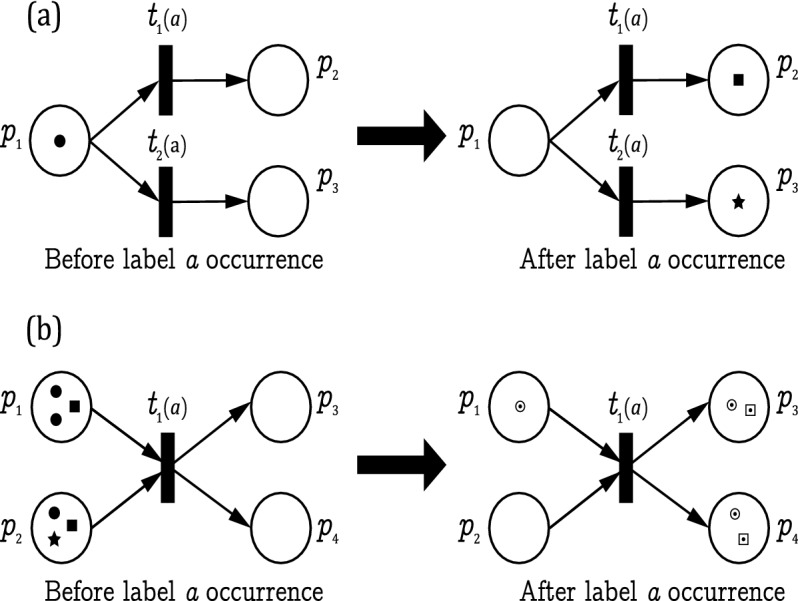
Observer net $\Phi$ dynamics.

Examine the LPN systems shown in Fig. 7 that illustrates the dynamics of the observer net by two different scenarios. In Fig. 7a, the initial state is given as $M_{\Phi ,0}=\{(p_1, \bullet )\}$, where $E=\{a\}$. Transitions $t_1$ and $t_2$ have the same label *a*.

Initially, based on Rule 1, transitions $t_1$ and $t_2$ are simultaneously enabled wrt color $\bullet$ at $M_{\Phi ,0}$ since $\Sigma ((p_1, \bullet ), a)=\{t_1,t_2\}$.

Then, based on Rule 2, the observer net simultaneously fires $t_1$ and $t_2$ since both are simultaneously enabled. The token of $(p_1, \bullet )$ is consumed by the execution of firing both transitions $t_1$ and $t_2$, which yields to a new reachable state composed of two colored markings $M_{\Phi ,1}=\{(p_2,\blacksquare ),(p_3,\bigstar )\}$.

In Fig. 7b, the initial state is given as $M_{\Phi ,0}=$ $\{(2p_1+p_2, \bullet ),$ $(p_1+p_2,\blacksquare ),$ $(p_2,\bigstar )\}$, where $E=\{a\}$.

Based on Rule 3, transition $t_1$ is recursively enabled wrt $\bullet$ and $\blacksquare$ at $M_{\Phi ,0}$ since we have $\Sigma ((2p_1+p_2, \bullet ), a)=\{t_1\}$ and $\Sigma ((p_1+p_2, \blacksquare ), a)=\{t_1\}$.

Then, based on Rule 4, the observer net recursively fires $t_1$, which yields to a new reachable state composed of two colored markings $M_{\Phi ,1}=\{(p_1+p_3+p_4,\odot ),(p_3+p_4,\boxdot )\}$.

According to Rule 5, the colored marking $(p_2,\bigstar )$ does not enable transition $t_1$. Thus the token with color type $\bigstar$ is removed from $p_2$, i.e., colored marking $(p_2, \bigstar )$ is discarded.

### Example 4

**Figure 8:**
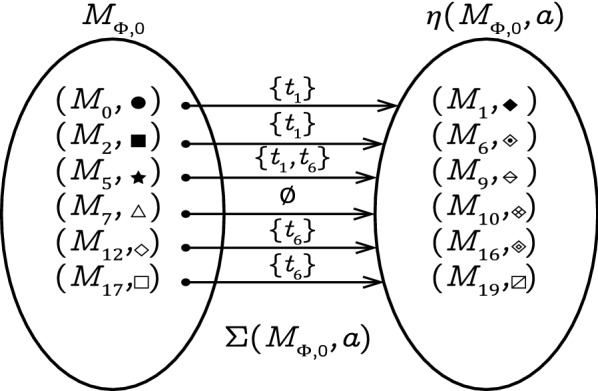
The set of markings reachable from $M_{\Phi ,0}$ by firing transitions with label *a*.

The observer net $\Phi$ of the LPN *G* in Fig. 1, is portrayed in Fig. 5. The initial state of the observer net is $M_{\Phi ,0} =$ $\{(M_0,\bullet ),$ $(M_2,\blacksquare ),$ $(M_5,\bigstar ),$ $(M_7,\triangle ),$ $(M_{12},\diamond ),$ $(M_{17},\square )\}$. Figure 8 shows the set of markings reachable from $M_{\Phi ,0}$ by firing transitions labeled with *a*, which is denoted as $\eta (M_{\Phi ,0}, a)$. Based on Rules 3 and 4, transition $t_1$ is recursively enabled at $(M_0,\bullet ), (M_2,\blacksquare )$, and $(M_5,\bigstar )$. In this case, the recursive firing of $t_1$ yields new colored markings $(M_1, {\mathcal {C}}_M(M_1))$, $(M_6,{\mathcal {C}}_M(M_6))$, $(M_9,{\mathcal {C}}_M(M_9))$. Similarly, transition $t_6$ is recursively enabled at $(M_5,\bigstar )$, $(M_{12},\diamond )$ and $(M_{17},\square )$. When firing, markings $(M_{10}, {\mathcal {C}}_M(M_{10}))$, $(M_{16}, {\mathcal {C}}_M(M_{16}))$, and $(M_{19}, {\mathcal {C}}_M(M_{19}))$ are reached. We can also say that $t_1$ and $t_6$ are enabled and fired simultaneously following Rules 1 and 2. Besides, we notice that the colored marking $(M_7, \triangle )$ does not enable any observable transition, i.e., $\Sigma ((M_7, \triangle ),e)=\emptyset$ for all $e \in E$, therefore tokens of $(M_7, \triangle )$ can be removed from the observer net based on Rule 5.

We define the unobservable reach of a state $M_\Phi$ as

5 $$\begin{aligned} \begin{aligned} {\mathcal {U}}(M_{\Phi }) = \left\{ (M',c')| (M,c)\in M_{\Phi }: M' \in {\mathcal {U}}(M)\right. \left. \text { and } c'={\mathcal {C}}_M(M') \right\} \end{aligned} \end{aligned}$$

A state transition function of $\Phi$ is given by : $\delta :~2^{M_{\Phi }}\times E \rightarrow 2^{M_{\Phi }}$ such that:

6 $$\begin{aligned} \delta (M^\prime _{\Phi }, e)= {\left\{ \begin{array}{ll} {\mathcal {U}}(\eta (M'_{\Phi }, e)), &{}\quad \text {if}~~\Sigma (M'_{\Phi }, e) \ne \emptyset \\ M'_{\Phi }, &{}\quad \text {otherwise.} \end{array}\right. } \end{aligned}$$

We call the set of states reachable from $M_{\Phi ,0}$ the reachable state set of $(N, M_{\Phi ,0})$, denoted by $R(N,M_{\Phi ,0})$.

### Example 5

Examine the LPN *G* in Fig. 1 and its associated observer net $\Phi$ in Fig. 5. The initial state of $\Phi$ is $M_{\Phi ,0}=$ $\{(M_0,\bullet ),$ $(M_2,\blacksquare ),$ $(M_5,\bigstar ),$ $(M_7,\triangle ),$ $(M_{12},\diamond ),$ $(M_{17},\square )\}$. The state estimation after observing event *a* from $M_{\Phi ,0}$ is given by the state transition function $\delta$ as follows:

$$\begin{aligned} \begin{aligned} M'_{\Phi } =\delta (M_{\Phi ,0}, a)= { {\mathcal {U}}( \eta (M_{\Phi ,0}, a)) =} \{(M_{1},\boxdot ),(M_{4},\boxminus ),(M_{6},\boxplus ), (M_{9},\odot ),(M_{10},\ominus ),(M_{11},\oplus ), (M_{15},\boxtimes ),(M_{16},\otimes ),(M_{19},\circledast )\} \end{aligned} \end{aligned}$$

**Figure 9:**
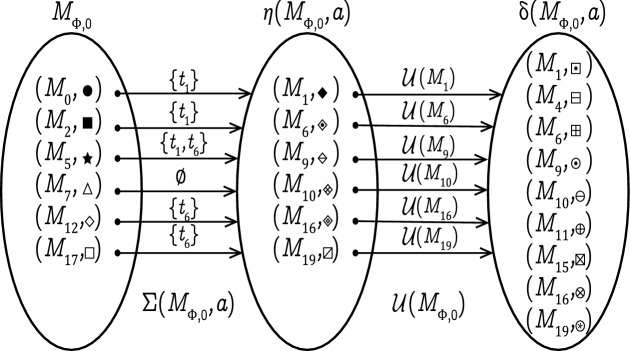
State transition from $M_{\Phi ,0}$.

The transition firing steps from $M_{\Phi ,0}$ that enable the occurrence of event *a* are illustrated in Fig. 9.

We define the operator $\llbracket . \rrbracket$ on the states of an observer net by $\llbracket M_{\Phi } \rrbracket =\{M \in {\mathbb {N}}^m| (M,c) \in M_{\Phi }\}$. As usual, the state transition function of $\Phi$ can be naturally extended to be $\delta :~2^{M_{\Phi }}\times E^* \rightarrow 2^{M_{\Phi }}$.

### Proposition 1

*Given an LPN* $G = (N,M_{0}, E, \ell )$ *and its observer net* $\Phi =(N,M_{\Phi ,0}, E, \ell , {\mathcal {C}}_c, {\mathcal {C}}_M)$, *we have* ${\mathcal {C}}(w)= \llbracket \delta (M_{\Phi ,0}, w) \rrbracket$ *for all* $w \in {\mathcal {L}}(N,M_0)$.

### Proof

We do so by mathematical induction on the length of the observation sequence *w*.

(Basis case) For $w=\varepsilon$, we have ${\mathcal {C}}(\varepsilon )={\mathcal {U}}(M_0)$ and $\delta (M_{\Phi ,0},\varepsilon )=M_{\Phi ,0}$. Since $\llbracket M_{\Phi ,0}\rrbracket = {\mathcal {U}}(M_0)={\mathcal {C}}(\varepsilon )$, then the result is clearly true.

(Inductive case) Assume that it is true for *w*. Then, we prove that it also holds for $w'= we$, where $e\in E$.

1. We have $${\mathcal {C}}(we) = \{M\in {\mathbb {N}}^m|$$ $$M''\in {\mathcal {C}}(w), t \in T_o: M''[t\rangle M', M \in {\mathcal {U}}(M') \text { and } \ell (t)=e\}$$.
2. By $\delta (M_{\Phi ,0}, we) = \delta (\delta (M_{\Phi ,0}, w), e)$, let $\delta (M_{\Phi ,0}, w)=M'_{\Phi }$. Then, it holds
  $$\delta (M_{\Phi ,0}, we) = \delta (M'_{\Phi }, e)$$.
3. Based on (6), it holds
  $$\delta (M'_{\Phi }, e) = {\mathcal {U}}(\eta (M'_{\Phi }, e))$$.
4. Based on (5), we have
  $$\delta (M'_{\Phi }, e) = \{(M,c)| (M',c') \in \eta (M'_{\Phi }, e): M \in {\mathcal {U}}(M') \text { and } {\mathcal {C}}_M(M')=c'\}$$.
5. Based on (4), one gets
  $$\begin{aligned} \delta (M'_{\Phi }, e) &=\{(M,c)| (M'',c'')\in M'_{\Phi }, (M',c')\in \eta ((M'',c''), e): M\in {\mathcal {U}}(M')\text { and }{\mathcal {C}}_M(M')=c\} {\text{ and}}\\ \delta (M'_{\Phi }, e) = \{(M,c)| (M'',c'')\in M'_{\Phi }, t \in \Sigma ((M'',c''), e): M''[t\rangle M', M \in {\mathcal {U}}(M')\text { and } {\mathcal {C}}_M(M')=c\} \end{aligned}$$.
6. Based on (2), it holds
  $$\delta (M'_{\Phi }, e) = \{(M,c)|(M'',c'')\in M'_{\Phi }, t\in T_o: M''[t\rangle M', M\in {\mathcal {U}}(M'), {\mathcal {C}}_M(M')=c\text { and }\ell (t)=e\}$$.

Combining (1) and (6), it holds ${\mathcal {C}}(we)= \llbracket \delta (M_{\Phi ,0}, we) \rrbracket$. $\square$

### Proposition 2

*Given an LPN* $G = (N,M_{0}, E, \ell )$, *its observer net* $\Phi =(N,M_{\Phi ,0}, E, \ell , {\mathcal {C}}_c, {\mathcal {C}}_M)$ *and a secret* $S\subseteq R(N,M_0)$, *an observation* $w\in {\mathcal {L}}(N,M_0)$ *is non-CSO with respect to* *S* *iff* $\llbracket \delta (M_{\Phi ,0}, w)\rrbracket \subseteq S.$

### Proof

(If) Suppose that $\llbracket \delta (M_{\Phi ,0}, w)\rrbracket \subseteq S$. Based on Proposition 1, we have $\llbracket \delta (M_{\Phi ,0}, w)\rrbracket = {\mathcal {C}}(w)$. According to Definition 3, ${\mathcal {C}}(w)\subseteq S$ holds and thus *w* is not-CSO.

(Only if) Let $w \in {\mathcal {L}}(N, M_0)$ be a non-CSO observation. Based on Definition 3, ${\mathcal {C}}(w)\subseteq S$ holds. Since we have already shown that $\llbracket \delta (M_{\Phi ,0}, w)\rrbracket = {\mathcal {C}}(w)$ in Proposition 1, then $\llbracket \delta (M_{\Phi ,0}, w)\rrbracket \subseteq S$ holds. $\square$

### Proposition 3

*Given an LPN* $G = (N,M_{0}, E, \ell )$, *its observer net* $\Phi =(N,M_{\Phi ,0}, E, \ell , {\mathcal {C}}_c, {\mathcal {C}}_M)$, *and a secret* $S\subseteq R(N,M_0)$, *the system* *G* *is non-CSO iff at least an observation* *w* *exists such that* $\llbracket \delta (M_{\Phi ,0}, w)\rrbracket \subseteq S.$

### Proof

It is inferred directly from Definition 4 and Proposition 2. $\square$

## Verification of current-state opacity

Next, methods for checking CSO of a DES in on-line and off-line settings are developed using the presented observer net model.

### On-line verification

This subsection presents an on-line algorithm devoted to verifying CSO for a given LPN system using an observer net. The observer net $\Phi$ of the plant *G* provides a state estimation of *G* after an observable event occurs, and then verifies the CSO of the system. A discussion on Algorithm 2 is presented next.
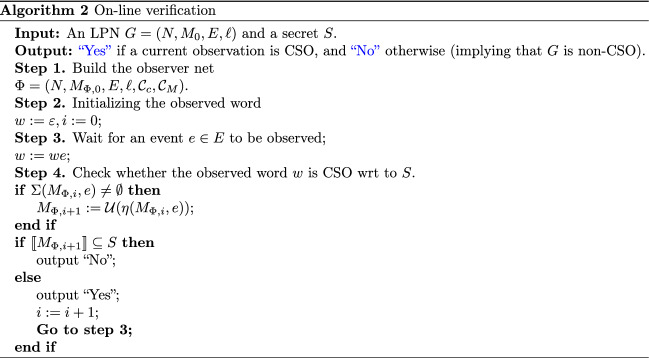


Algorithm 2 takes an LPN $G = (N,M_{0}, E, \ell )$ and a secret *S* as input. For any current observation *w*, the algorithm checks its CSO and returns “Yes” if it is opaque; otherwise it returns “No”, meaning that *G* is non-CSO. The first step builds the observer net $\Phi$ for *G*. Then, we initialize the observed word *w* in the second step. Upon observing the occurrence of any event $e\in E$, *w* is updated in Step 3. Then, we check its CSO with respect to *S* in Step 4. Specifically, when an event *e* occurs at $M_{\Phi , i}$, the observer net $\Phi$ generates a new state $M_{\Phi ,i+1}$ by simultaneously and recursively firing the enabled transitions with label *e*. If the set of colored markings in $M_{\Phi ,i+1}$ is not fully included in the secret *S*, then, by Proposition 1 and Definition 1, *w* is CSO. In this case, it executes Step 2 and waits for the occurrence of a new event; otherwise, *w* is non-CSO, and the opacity property is violated according to Proposition 2. Consequently, based on Proposition 3, *G* is non-CSO with respect to *S*.

Algorithm 2 employs the observer net for CSO verification. After an event occurrence, we need to compute the state estimation and then check if the opacity condition holds. Although the algorithm has the exponential space complexity in the worst case, compared with the RG-based verification approach, the main advantage of the on-line verification consists in limiting the analysis to the observed word only instead of exploring the whole language generated by the PN. Besides, the concept of simultaneous and recursive transition firing permits the concurrent execution of transition sequences and results in a significant decrease in the time complexity compared with the classical off-line opacity verification.

#### Example 6

Apply Algorithm 2 to LPN *G* shown in Fig. 1 and its associated observer net $\Phi$ depicted in Fig. 5 whose initial state is $M_{\Phi ,0} =$ $\{(M_0,\bullet ),$ $(M_2,\blacksquare ),$ $(M_5,\bigstar ),$ $(M_7,\triangle ),$ $(M_{12},\diamond ),$ $(M_{17},\square )\}$. Given a secret $S = \{M_{8},M_{13},$ $M_{14},M_{18},$ $M_{20}\}$, in the following, we show how Algorithm 2 operates if an observation $w = aba$ occurs.

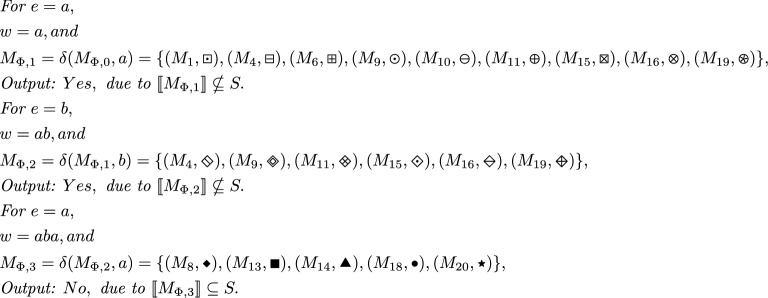



By applying Algorithm 2 to plant *G*, given $w=a$, we obtain $\llbracket M_{\Phi ,1} \rrbracket \nsubseteq S$. Then the observation $w=a$ is CSO with respect to *S*. After that, *w* is updated and the observer net $\Phi$ computes $M_{\Phi ,2}$ and also we have $\llbracket M_{\Phi ,2} \rrbracket \nsubseteq S$, indicating that the observation $w = ab$ is CSO with respect to *S*. Finally, when event *a* occurs, the on-line algorithm outputs “No” since $\llbracket M_{\Phi ,3} \rrbracket \subseteq S$ holds, implying that the observation $w=aba$ is not opaque with respect to *S*. According to Proposition 3, the LPN system *G* is non-CSO wrt *S*.

If Algorithm 2 never outputs “No”, we infer that all the previously generated observations are CSO. Once Algorithm 2 returns “No”, based on Definition 4, we conclude that *G* is non-CSO.

### Off-line verification

Next, we develop Algorithm 3 to construct the RG of an observer net, called a colored estimator, which can be used for the purpose of an off-line CSO verification.
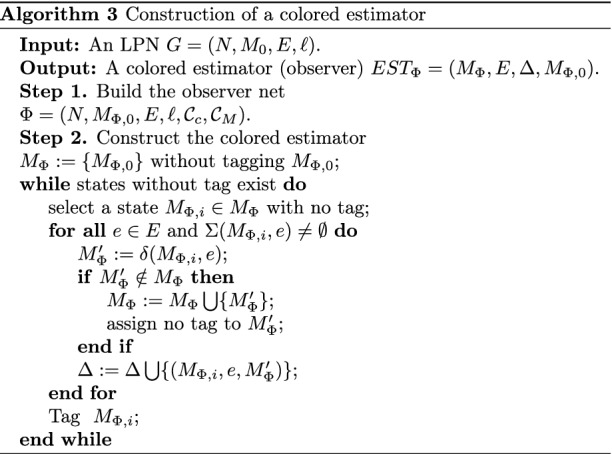


Let us now clarify how Algorithm 3 works. It takes $G = (N,M_{0}, E, \ell )$ as input and outputs an automaton $EST_{\Phi }=$ $(M_{\Phi },E,$ $\Delta ,M_{\Phi ,0})$, also called an observer or a colored estimator. Initially, we build the observer net $\Phi$ of *G*. In the second step, we start constructing the colored estimator by initializing the set $M_\Phi$ to ${M_{\Phi ,0}}$. Then, for all states $M_{\Phi , i} \in M_{\Phi }$ that have not yet been explored (i.e., without tags) and all events $e\in E$, we check if the set of enabled transitions $\Sigma (M_{\Phi ,i}, e)$ is not empty. Then we move to the next state computation. This procedure runs iteratively until all states in $M_{\Phi }$ are explored. Each state in $M_{\Phi }$ represents set of markings consistent with observation. In the worst-case scenario, Algorithm 3 can compute the whole reachability set, suggesting that the space complexity can grow exponentially with the number of tokens at the initial marking. Algorithm 3, on the other hand, exploits the efficient mechanism of the observer net $\Phi$, namely, simultaneous and recursive transition firing, to generate a straightforward state-estimator without constructing the reachability graph of *G*. The complexity analysis of the algorithm shows a reduced time complexity compared with other related works. Numerical results for approving this benefit are given in section “Computational complexity and comparison”.

#### Proposition 4

*Given an LPN* $G = (N,M_{0}, E, \ell )$, *its observer net* $\Phi =(N,M_{\Phi ,0}, E, \ell , {\mathcal {C}}_c, {\mathcal {C}}_M)$, *and a secret* $S\subseteq R(N,M_0)$, *iff each state* $M_{\Phi , i}$ *of the colored estimator obtained by Algorithm 3 is not fully included in* *S*, *i.e.,* ${\llbracket M_{\Phi ,i}\rrbracket } \nsubseteq S$, *then the system is current-state opaque wrt* *S*.

#### Proof

From Proposition 1, we have ${\mathcal {C}}(w)= \llbracket \delta (M_{\Phi ,0}, w)\rrbracket$. According to Definition 4, if we find at least a state $M_{\Phi ,i}$ of the colored estimator that satisfies $\llbracket M_{\Phi ,i} \rrbracket \subseteq {\mathcal {S}}$, then the LPN is non-CSO wrt *S*; otherwise, the system is CSO. $\square$

#### Example 7

**Figure 10:**
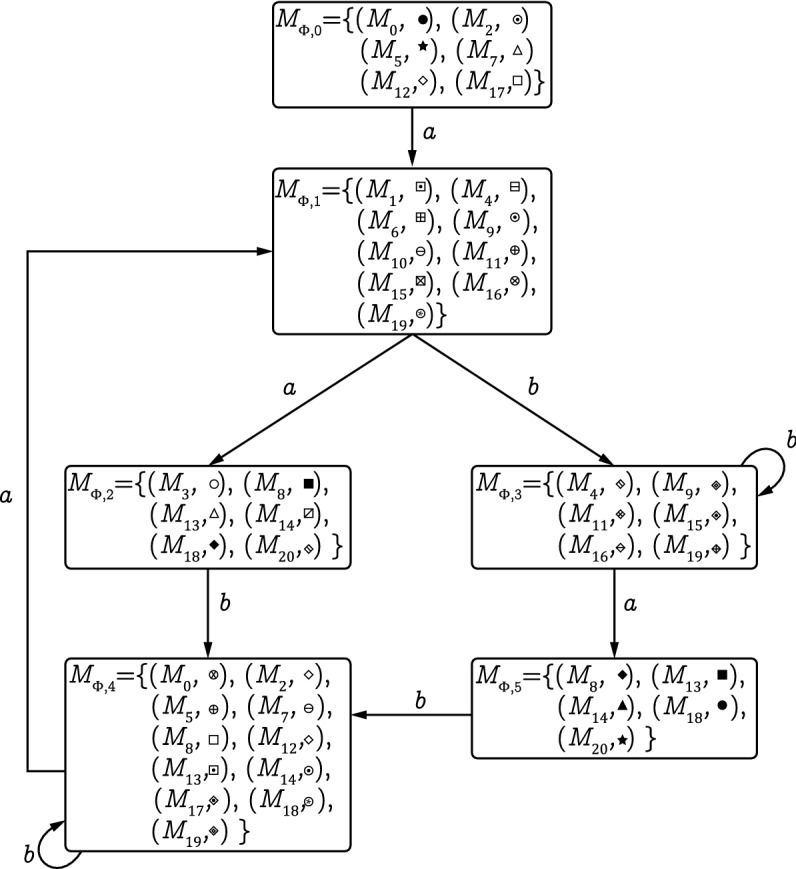
The colored estimator of the observer net.

Consider the LPN system *G* shown in Fig. 1. Its observer net $\Phi$ is depicted in Fig. 5. The colored estimator of *G*, generated by Algorithm 3, is shown in Fig. 10. Let $S = \{M_{8},M_{13},M_{14},M_{18},M_{20}\}$ be a secret. We have $\llbracket M_{\Phi ,5}\rrbracket \subseteq S$, it follows from Proposition 4 that the LPN is non-CSO with respect to *S*.

## Computational complexity and comparison

The effectiveness of the approach developed in section “Verification of current-state opacity” is investigated in this section by comparing it with the opacity verification methods recently proposed in the literature to demonstrate their advantages and limitations. It is based on the CPU time in seconds of a desktop computer running under the operating system Windows 10 with I7.4 CPU 3.40 GHz, 32 GB memory.

To do so, we apply the proposed on-line algorithm to a larger version of the LPN in Fig. 1, where the initial marking is $M_0=15p_1$. Let $S=\{(4p_3+11p_5), (5p_1+10p_5), (8p_1+3p_5+4p_6), (11p_1+2p_2+2p_3), (13p_1+2p_5), (14p_1+p_5), (15p_1)\}$ be a secret. We use the standard opacity verification approach, which consists of computing the RG and converting the obtained RG into its equivalent DFA. After that, for each state of the observer (i.e., ${\mathcal {C}}(w)$), we check whether it is fully included in the secret or not. This method takes more than $1.9 \times 10^4$ seconds and shows that the considered LPN system is non-CSO. This computational overhead is mainly caused by the RG construction and conversion from an NFA to a DFA.

**Table 1:** Performance of Algorithm 2.

Event occurrence	Time (s)	CSO
Observable event *a* occurs	$7.4 \times 10^{-2}$	Y
Observable event *a* occurs	$6.7 \times 10^{-1}$	Y
Observable event *b* occurs	$1.1 \times 10^{1}$	Y
Observable event *a* occurs	$2.6 \times 10^{1}$	Y
Observable event *a* occurs	$3.9 \times 10^{1}$	Y
Observable event *b* occurs	$5.7 \times 10^{1}$	N

Now, let us implement the on-line algorithm to the same example. Table 1 shows the performance of Algorithm 2. The first column represents the occurrence of an event. The second indicates the time (CPU seconds) required to run an observer net when an event occurs. The third column shows the algorithm’s outputs when an event occurs: “Y” if the observation is CSO and “N” otherwise. From Table 1, it is known that the observed event sequence $\textit{w = aabaab}$ is non-current-state opaque wrt *S*. Thus, by Definition 4, the LPN is non-CSO with respect to the given secret. Consequently, due to the short time taken to verify if an observation is CSO or non-CSO, we conclude that the proposed approach can be used for real-time verification. However, this process can be computationally intensive, mainly when an observed word’s length is excessively long.

**Table 2:** Performance of Algorithm 3.

*k*	|*RG*|	$|Obs_{RG}|$	Time (s)	$|EST_{\Phi }|$	Time (s)
2	21	6	$0.8 \times 10^{1}$	6	$0.2\times 10^{1}$
4	126	15	$1.4 \times 10^{2}$	15	$0.4\times 10^{2}$
8	1287	39	$4.7 \times 10^{3}$	39	$1.4\times 10^{3}$
10	3003	–	o.t.	54	$5.2\times 10^{3}$
20	53,130	–	o.t.	159	$1.0 \times 10^{4}$
40	–	–	o.t.	–	o.t.

Examine the LPN system *G* in Fig. 1 with initial marking $M_0=kp_1$, where $k\in {\mathbb {N}}$. Accordingly, we consider a family of nets rather than a single LPN, which is parameterized by the initial marking. Table 2 compares the colored estimator construction using the observer net, i.e., $EST_{\Phi }$, as shown in Algorithm 3, with the standard approach for observer construction, i.e., DFA construction using the RG of an LPN. The first column shows the value of *k*. The number of reachable markings is represented in Column 2. Columns 3 and 4 give the number of states of the standard observer and its construction time, respectively. Finally, Columns 5 and 6, respectively, expose the number of states in the observer net and the time of its construction. We use the notation “o.t.” (out of time) to indicate that the computation takes more than three hours to complete. Both methods are computationally demanding in the worst case. However, the observer net’s advantage compared with the standard approach consists of a lower time cost and simplicity of construction.

**Table 3:** Observer net for current-state opacity verification.

	Framework	Acyclic	On-line/Off-line	NFA to DFA	Complexity
^10^	NFA	Yes	No	Yes	PSPACE-complete
^27^	LPN	Yes	No	Yes	PSPACE-complete
^28^	POPN	Yes	No	No	PSPACE-complete
Our work	LPN	Not required	Yes	No	PSPACE-complete

Table 3 exposes the proposed observer net’s advantages compared with the recent works for the opacity verification problem. For this purpose, we choose three typical methods respectively presented in^10,27,28^. The second column indicates the application framework. The third column shows the presence of an acyclicity assumption. Column 4 indicates whether the method applies to on-line and off-line settings. Column 5 shows whether we need a conversion from an NFA to a DFA. Finally, Column 6 shows the complexity of each method. Notice that the proposed approach outperforms the related works by getting rid of the acyclicity assumption; in addition, it provides a straightforward strategy for the estimator construction by avoiding the conversion from NFA to DFA. In the worst case, it has an exponential space complexity as it is possible to compute all the reachable markings. However, the observer net has a lower practical time overhead compared with related works due to using the new concepts of simultaneous and recursive transition enabling and firing.

## Conclusions

This paper proposes a new PN subclass, called an observer net, to verify the current-state opacity of a DES modeled with an LPN. We define the structure and dynamics (transition enabling and firing rules) for the observer net, which is useful in providing a rapid computation of the set of markings consistent with each observation. Then, we consider on-line and off-line settings for opacity verification. In the on-line setting, the proposed algorithm observes the event occurrence and then decides on-line if the observed word is CSO with respect to the predefined secret. In the off-line setting, we design an algorithm for constructing a colored (state) estimator used for opacity verification rather than the conventional methods based on computing the state estimate (i.e., the set of consistent markings) by constructing a DFA from the reachability graph of a plant.

In the proposed model, every node of the colored estimator corresponds to the consistent markings, making the reachability graph construction unnecessary. Finally, a comparison study is conducted to validate the effectiveness of this approach. Our future work includes exploring other security problems and potential vulnerabilities such as unauthorized access, cyberattacks, intrusion detection, prevention, etc.

## Data Availability

The datasets generated and analysed during the current study are available in the GitHub repository, https://github.com/LabedAJalil/Observer-net.
